# Exploring the paradox: double burden of malnutrition in rural South Africa

**DOI:** 10.3402/gha.v6i0.19785

**Published:** 2013-01-24

**Authors:** Peter A. Cooper

**Affiliations:** Department of Paediatrics and Child Health, Charlotte Maxeke Johannesburg Academic Hospital, University of the Witwatersrand, Johannesburg, South Africa

Growth during intrauterine life, infancy, childhood, and adolescence has a powerful effect on morbidity and mortality at each of these stages of life and the effects persist for the remainder of the individual's life. For many decades, it has been recognized that infectious diseases have been the leading cause of death in children under the age of 5 in developing countries. It is only in recent years that the potentiating effects of undernutrition on infectious diseases have been recognized. Data analyzed from developing countries showed that more than half of child deaths were attributed to the potentiating effects of undernutrition and, of these, 80% were attributable to mild–moderate undernutrition rather than severe undernutrition (1). Thus, high rates of undernutrition in young infants are of great concern. Increasing rates of being overweight and obese in later childhood and adolescence in developed countries have been noted for several decades. However, in low- and middle-income countries (LMIC), where high rates of undernutrition in young infants still exist, this has only become the focus of attention in more recent years giving rise to the concept of a double burden of malnutrition in LMIC.

The study by Dr. Kimani-Murage in a poor rural area of South Africa, a middle-income country, confirms that this double burden is now well established even in this setting. The rate of stunting was high in this study peaking at 32% at 1–2 years of age and reflects the effects of inadequate growth during intrauterine life and during the first year. This study did not look at infants under the age of 1 year, but large cohort studies in South Africa and other LMIC have shown an increase in the rates of stunting from birth to 2 years of age. It is well known that the rates of exclusive breast-feeding up to the age of 6 months are low in most parts of the developing world and particularly low in South Africa. This and inadequate diets beyond 6 months of age are largely responsible for this age-related increase in stunting. The overall figure for stunting of 18% for those <5 years of age is however approximately 30% lower than the figures for rural South Africa found during studies in the 1990s, suggesting that there is a downward trend in the prevalence of stunting in the second decade after the emergence of a democratic government. However, these rates are still high and the known associations of greater morbidity and mortality in childhood make it imperative that effective strategies be implemented to reduce these rates. In addition, growth failure up to 12 months of age is strongly associated with reduced adult stature and the concomitant adverse effects on adult health and performance.

This study was carried out in South Africa before the introduction of more effective programs in preventing mother-to-child transmission (PMTCT) of HIV and the rollout of antiretroviral therapy for those infected with HIV. In this study, the relatively low number of children infected with HIV (4.4%) was probably a reflection of the high mortality of HIV-infected infants under the age of 1 at the time of this study as only those >1 year of age were included. What is noteworthy is the high acceptance rate of 95% for testing of children for HIV and the generally favorable effects on the care of those children who tested positive even at a time when effective antiretroviral therapy was not readily available. The nutritional status of those infected with HIV was poorer than those uninfected. With a far more effective PMTCT program now in place and the widespread availability of antiretroviral therapy, the overall contribution of HIV to undernutrition in children under the age of 5 should have decreased significantly since this study was done.

Low birth weight is well recognized as a risk factor for adult glucose intolerance. However, while some studies have suggested that catch-up growth during the early years after birth may have a detrimental effect on glucose tolerance later on, in large birth cohort studies in LMIC, catch-up in weight up to 48 months of age was not shown to be a risk factor for adult glucose intolerance. This suggests that this time frame may provide a window of opportunity when improved nutrition may result in better childhood survival and improved adult human capital without increasing the risk for adult diabetes (2). However, there is strong evidence from these and other studies that the development of overweight and obesity in later childhood and adolescence is a strong risk factor for later glucose intolerance and diabetes.

Studies from around the world have shown a dramatic increase in childhood and adolescent obesity in recent decades resulting in what has been termed a global epidemic. Most of the data has come from developed countries and urbanized parts of some LMIC. This study has shown a prevalence of overweight and obesity in a poor rural area of South Africa, mainly among girls, in the second decade of life, comparable with data from developed countries. The prevalence in girls who had reached Tanner stage 5 of puberty was 35% and this figure rises to greater than 50% in black adult females in South Africa. There are minimal data from other parts of sub-Saharan Africa regarding overweight and obesity in adolescence, but the pattern seen in this study is similar to that in LMIC in other parts of the world such as Latin America and Asia. The difference in prevalence of overweight and obesity between boys and girls in this study has been seen in a number of other studies including those in South Africa. Dr. Kimani-Murage suggests some of the factors responsible for this difference including less physical activity and more problematic eating habits in adolescent girls, but this is clearly an area in need of in-depth research. Less than 5% of adolescent boys were overweight or obese in this study, but the figure for adult black males in South Africa is at least 25% indicating that the onset of obesity in males begins in adulthood rather than adolescence. As mentioned, obesity in adolescence and adulthood is strongly associated with later diabetes, but it is also associated with other diseases such as hypertension, renal, and cerebrovascular disease. A further concern is the finding in a recent analysis of 27 national data sets from countries in sub-Saharan Africa that maternal obesity during pregnancy is associated with an increased risk of early neonatal death (3). These high levels of obesity will have major implications for the health system in decades to come.

South Africa is economically much better off than most other sub-Saharan African countries. However, many sub-Saharan African countries have shown impressive economic growth during the past decade and, although the under-5 mortality rate is unlikely to achieve the Millennium Development Goal of a two-thirds reduction from the baseline in 1990–2015, the latest figures from UNICEF show an impressive reduction in the under-5 mortality rate in sub-Saharan Africa from 178 per thousand live births in 1990 to 109 per thousand live births in 2011—most of this reduction taking place after 2000. Previous studies in developing countries have shown that in countries with very low gross domestic product (GDP), the increase in overweight adult females over two recent decades has been mainly in those socioeconomically better-off groups compared with those countries with higher GDP, where the increase has been greater in the lowest socioeconomic groups (4). South Africa, as a middle income country, appears to be following the latter pattern at this stage. However, with improved economic growth and a rapidly falling under-5 mortality rate, it is likely that many countries in sub-Saharan Africa will soon follow this pattern resulting in a rapid rise in adolescent and adult obesity in those countries. There may still be a window of opportunity to intervene in low-income countries throughout the world to modify this expected increase in obesity.
